# Editorial: The Brain – Gut – Microbiome Network in Metabolic Regulation and Dysregulation

**DOI:** 10.3389/fendo.2021.760558

**Published:** 2021-09-10

**Authors:** Pierre De Meyts, Nathalie Delzenne

**Affiliations:** ^1^de Duve Institute, Catholic University of Louvain, Brussels, Belgium; ^2^Stem Cell Research Department, Novo Nordisk A/S, Måløv, Denmark; ^3^Louvain Drug Research Institute, UCLouvain, Brussels, Belgium

**Keywords:** gut microbiota, gut virome, gut bacteriophages, glycemic control, obesity, type 1 and type 2 diabetes, non-alcoholic fatty liver disease, pre/probiotics

The very nature of endocrinology has undergone major paradigm shifts over the last few decades with the discovery of endocrine functions by organs not hitherto considered as endocrine organs, such as the adipose tissue, the gut and the brain. Those organs release hormones and bioactive compounds acting as metabolism regulators and involved in organ crosstalk. In addition, a major conceptual revolution has taken place in the last few years with the explosion of interest in the role of the symbiosis between eukaryotic cells and the microbes hosted by the animal and human body (microbiome), primarily in the gut [(1); Gérard and Vidal]. The gut microbiome is now recognized as a real functional organ which influences host physiology and plays important roles in digestion, nutrition, immune regulation and metabolism. The organ crosstalk between the brain and the gut including its microbiome was the theme of the 43^rd^ Symposium on Hormones and Cell Regulation held in Mont Ste Odile in Alsace, France, October 10-13, 2018, organized by Nathalie Delzenne and Pierre De Meyts, entitled “The Brain - Gut - Microbiome Network in Metabolic Regulation and Dysregulation”, sponsored by the European Society of Endocrinology. This Frontiers Research Topic with the same title contains four reviews based on lectures given at the Symposium.

The review by Gérard and Vidal discusses the impact of gut microbiota on host glycemic control and its potential implications for the treatment of obesity and type 2 diabetes mellitus (T2DM). As they mention, obesity represents the fifth leading cause of death in the world, accounting for almost 3.4 million deaths each year. It is predicted that 38% of the world’s adult population will be overweight and another 20% obese by the year 2030 if the current trend continues. The prevalence of diabetes (more than 90% being T2DM) is increasing also around the world, reaching 425 million in 2017, and resulting in 5 million deaths that year. Thus, the development of efficient prevention and therapeutic interventions is an imperative global public health issue. In that context, the review addresses how gut microbiota affect and regulate key metabolic functions such as glucose regulation and insulin resistance. The authors examine key biological molecular mechanisms underlying the impact of gut microbiota on host glycemic control including: insulin secretion, short-chain fatty acid production, bile acid metabolism, and adipose tissue regulation. They highlight how prebiotic/probiotic interventions affect these bacterial processes and are now considered as promising approaches to treat obese and diabetic patients. They mention however how the lack of well-defined and standardized methodology to identify and select compounds and bacterial strains with proven antidiabetic properties results currently in a low efficacy of pre/probiotic strategies. This is probably the challenge of the next 5 years, suggest the authors.

Besides T2DM, another widespread co-morbidity of obesity is non-alcoholic fatty liver disease (NAFLD), the most common liver disease worldwide (2). It may evolve into its inflammatory complication i.e. non-alcoholic steatohepatitis (NASH), liver cirrhosis and hepatocellular carcinoma (Grabherr et al.). Here also, certain host factors and particularly the gut microbiota are assumed to be involved in the evolution from NAFLD to NASH (Grabherr et al.). In this Research Topic, this involvement of “bacterial dysbiosis” is discussed in the review by Grabherr et al. in Herbert Tilg’s lab. NAFLD has been shown to contain a disease-specific gut microbiome signature: certain pro-inflammatory bacteria are dominantly present in these patients, while protective bacteria are decreased. Various bacterial metabolites and microbiota generated secondary bile acids are involved in NAFLD metabolic dysfunction. Further research is needed to provide evidence that interference at the level of the gut microbiome will be beneficial for treating NAFLD.

Although much attention has been given to the bacterial component of the human gut microbiome, viruses have been often overlooked although they are estimated to be abundant and have been associated with human disease (3). Bacteriophages (viruses that infect bacteria) constitute the majority of viral particles and impact microbial ecosystems processes through phage predation, lysogeny and horizontal gene transfer [(3); Sutton and Hill]. Despite their ubiquity, our knowledge of viral genomic diversity in the microbiome is limited, because most viral sequences fail to match existing genome databases [(3); Sutton and Hill]. In the next review of this Research Topic, Sutton and Hill state that understanding the mechanisms that underpin phage-host interactions is vital if we propose to use the virome as a diagnostic or therapeutic tool in the future. Our current understanding of phage host dynamics in the gastrointestinal tract is limited. The authors provide an extensive review of what is known about phage-host interactions. Progress will depend heavily on sequence-based approaches and *in silico* tools. The authors discuss critically the pros and cons of available methods, whether these created an unstable foundation, and how to provide a solid basis for future experimentation by developing robust research methods (Sutton and Hill).

The last review in this Research Topic by Dedrick et al. addresses the role of the gut microbiota and environmental factors in Type 1 diabetes pathogenesis. They provide a comprehensive review of the literature. Genome-wide association studies have identified ca 50 genetic regions that affect the risk of developing T1D. However, the increasing incidence rates, immigrant studies, and twin studies suggest that environmental factors play an important role and the trigger cannot simply be explained by genetic predisposition (Dedrick et al.). More recently, the interplay between the gut microbiota and the immune system has been implicated as an important factor in T1D pathogenesis. Longitudinal and cross-sectional human studies, comprehensively reviewed here, have revealed broad compositional and diversity patterns although results are not always concordant. T1D patients have a less diverse gut microbiota, an increased prevalence of Bacteriodetes taxa and an aberrant metabolomic profile compared to healthy controls (Dedrick et al.). Environmental factors, such as birth mode, diet and antibiotic use modulate the gut microbiota and contribute to T1D. Finally, the authors discuss how microbiota-produced metabolites, proteins and gut virome protect against or trigger T1D onset. They conclude that higher levels of diversity and the presence of beneficial microbes and microbial metabolites can protect against T1D onset. A full understanding of the interplay between host and microbes still remains elusive.

In conclusion, the four reviews summarized here represent a fair sample of the many splendid presentations and vivid discussions on the brain – gut – microbiome axis that took place at the Mont Ste Odile Symposium. They also stress the methodological barriers that remain to be overcome in order to progress towards an efficient pre/probiotic therapeutic strategy for metabolic disorders.

## Author Contributions

All authors contributed to the article and approved the submitted version.

## Conflict of Interest

Author PDM is an unpaid external consultant for Novo Nordisk A/S.

The remaining authors declare that the research was conducted in the absence of any commercial or financial relationships that could be construed as a potential conflict of interest.

## Publisher’s Note

All claims expressed in this article are solely those of the authors and do not necessarily represent those of their affiliated organizations, or those of the publisher, the editors and the reviewers. Any product that may be evaluated in this article, or claim that may be made by its manufacturer, is not guaranteed or endorsed by the publisher.
